# Decreased Human Leukocyte Antigen-G Expression by miR-133a Contributes to Impairment of Proinvasion and Proangiogenesis Functions of Decidual NK Cells

**DOI:** 10.3389/fimmu.2017.00741

**Published:** 2017-06-28

**Authors:** Wenwei Guo, Liang Fang, Bo Li, Xifeng Xiao, Shuqiang Chen, Jun Wang, Fang Yang, Lihua Chen, Xiaohong Wang

**Affiliations:** ^1^Department of Gynecology and Obstetrics, Tangdu Hospital, The Fourth Military Medical University, Xi’an, China; ^2^Department of Immunology, The Fourth Military Medical University, Xi’an, China

**Keywords:** miR-133a, human leukocyte antigen-G, decidua, nature killer cell, killer immunoglobulin-like receptor 2DL4, recurrent spontaneous abortion

## Abstract

Human leukocyte antigen (HLA)-G plays a crucial role in conferring fetal–maternal tolerance and ensuring a successful pregnancy. CD56^bright^ natural killer (NK) cells accumulate at the maternal decidua in large numbers during pregnancy and are found in direct contact with fetal trophoblasts. There are increasing evidences that decidual NK (dNK) cells are crucial for pregnancy. However, the regulation of dNK cells is mostly unknown. Here, we provide evidences that the secretion function of dNK cells in recurrent spontaneous abortion was impaired, which led to the impairment of the proinvasion and proangiogenesis functions of dNK cells. Decreased HLA-G expression induced by the transfection of miR-133a mimics in HTR-8/SVneo affected the secretory functions of dNK cells. Thus, our data revealed that the functions of dNK cells could be suppressed by the decreased expression of HLA-G and suggest a possible mechanism of recurrent miscarriage.

## Introduction

Recurrent spontaneous abortion (RSA) is defined as two or more consecutive spontaneous abortions with the same partner (1). However, the causes of RSA are complicated and mostly unknown. In addition to chromosomal abnormalities, anatomical anomalies, and endocrine disorders, immunologic dysfunction is generally considered the most important cause leading to RSA (2, 3). Whether gravid or inchoate, there is a unique combination of human leukocyte antigen (HLA) expression on extravillous trophoblasts (EVTs) and maternal leukocytes in decidual tissues at the maternal–fetal interface. EVT expresses the polymorphic non-classical HLA class I antigens HLA-E, HLA-F, HLA-G, and HLA-C (4, 5). Of these, HLA-G is the most frequently studied and plays important roles in inducing immune tolerance to maintain pregnancy (6–8).

In humans, approximately 40% of the decidual cells are leukocytes, and more than 70% are CD56^bright^CD16^−^ natural killer (NK) cells (9), which are a minority of the blood NK population. Only about 10% of lymphocytes are CD56^dim^CD16^+^ NK cells in decidual cells which are the main population in peripheral blood and display cytotoxic activity toward the EVTs (10). Compared to peripheral blood CD56^dim^CD16^+^ NK cells (pNK), CD56^bright^CD16^−^ NK cells have a higher secretory ability and lower cytotoxicity (11). Therefore, these CD56^bright^CD16^−^ NK cells are defined as decidual NK (dNK) cells (12). Compared to pNK cells, dNK cells express higher levels of certain inhibitory receptors that can recognize non-classical HLA class I antigens on EVTs (13). Then, these receptors transfer inhibitory signals to reduce the cytotoxicity of dNK cells (12). dNK cells also express CD94/NKG2A and killer immunoglobulin-like receptor (KIR) that could recognize HLA-C and HLA-G/E, respectively. The interaction between dNK cells and such HLA molecules would contribute to maintain pregnancy successfully (14). Recent studies have shown that dNK cells play an important role in early pregnancy (15–19). A report by Hanna et al. indicated that dNK cells possess the unique ability to regulate crucial placental developmental processes, including trophoblast invasion and vascular growth, *via* the production of interleukin (IL)-8, interferon induced protein (IP)-10, and vascular endothelial growth factor (VEGF) (15). Moreover, NK cells belong to the group 1 innate lymphoid cells (ILC1). The human decidua tissues also contain the group 3 ILC which express CD56 and NCR but lack CD94/NKG2A and KIR. These NCR^+^ ILC3 cells could also produce IL-8, which may be involved in the trophoblast invasion and neo-angiogenesis process (20, 21).

Killer immunoglobulin-like receptor 2DL4 (KIR2DL4), a specific receptor for HLA-G, is expressed on human NK cells (22). KIR2DL4 has an immunoreceptor tyrosine-based inhibitory motif (ITIM) and shows weak inhibition of dNK cell when bound to HLA-G. Therefore, it has been suggested that NK cells expressing KIR2DL4 might be involved in the maintenance of pregnancy by recognizing HLA-G (23, 24). The receptors of HLA-G also include immunoglobulin-like transcript 2 (ILT2) and ILT4. Both ILT2 and ILT4 contain three ITIMs and are inhibitory receptors expressed on leukocytes including NK cells (25).

MicroRNAs (miRNAs) are a class of non-protein-coding RNAs that are estimated to regulate 30% of all genes in animals by binding to specific sites in the 3′ untranslated region (UTR) (26, 27). In studying the mechanisms underlying RSA, we found that miR-133a was greatly overexpressed in RSA villi compared to villi from induced abortion (IA) patients. Multi-software prediction and real-time PCR confirmed that miR-133a was most likely to bind to the HLA-G 3′UTR, as established in our previous study (28). Therefore, this study was designed to confirm that miR-133a negatively regulates HLA-G expression to influence dNK function *via* KIR2DL4 in RSA patients.

## Materials and Methods

### Human Samples

Maternal decidual tissues were obtained from 12 patients with RSAs and from 11 patients with IA, excluding chromosomal and anatomic abnormalities as causes for abortion. Maternal peripheral blood mononuclear cells were prepared from six patients with IA and RSA. The normal and RSA samples were aged between 7 and 12 weeks of gestation, and all samples were obtained from the Tangdu Hospital of the Fourth Military Medical University. Written informed consent was obtained from each subject, and this study was approved by the Institutional Review Board, Tang Du Hospital, Fourth Military Medical University. The decidual tissues were placed into cold PBS immediately and quickly transported to the laboratory.

### Human Decidual Cell Isolation, dNK Purification, and Culture

For the isolation of decidual cells, an enzymatic dispersion method was used, as described previously (17, 29). Briefly, the decidual tissues were washed in PBS twice and then cut into small pieces. The decidual tissues were digested with 1 mg/ml collagenase type IV (MP Biomedical, Santa Ana, CA, USA) and 0.01 mg/ml DNase I (MP Biomedical, Santa Ana, CA, USA) in RPMI 1640 medium (HyClone Cell Culture, Carlsbad, CA, USA) in 37°C for 40 min. The suspensions were filtered through a nylon mesh, and the supernatants were discarded after centrifugation. The decidual mononuclear cells were isolated by density gradient centrifugation with Ficoll and were then immediately used for flow cytometry analysis and further study. CD56 Microbeads (Miltenyi Biotec, Bergisch Gladbach, Germany) were used for the positive selection of dNK cells from the decidual mononuclear cells. For excluding ILC3 cells, dNK cells were stained with PE-conjugated anti-CD94 antibodies at 4°C for 30 min for fluorescence-activated cell sorting (FACS), and >97% purity was obtained for CD56^+^CD16^−^CD94^+^ cells. These cells were used for the subsequent experiments. The CD56^+^CD94^+^ dNK cells were cultured in RPMI 1640 medium containing 10% fetal bovine serum (FBS; Gibco, Grand Island, NY, USA) and 100 IU/ml penicillin/streptomycin plus 20 ng/ml IL-15 (PeproTech, Rocky Hill, NJ, USA) for 24 h, and culture supernatants were harvested for the subsequent experiments. When CD56^+^CD94^+^ dNK cells were cocultured with HTR-8/SVneo cells, there were no IL-15 stimulation. Peripheral blood samples from IA and RSA were centrifuged (800 × *g*, room temperature) for 30 min on Ficoll gradients (MP Biomedical, Santa Ana, CA, USA) to collect mononuclear cells.

### Flow Cytometry

The following conjugated mouse anti-human antibodies were used for FACS analysis: CD45-APC, CD3-Percep/Cy5.5, CD94-Percp/Cy5.5, CD56-FITC, CD16-PE, NKp44-APC, NKp46-APC, NKG2A-APC, NKG2D-APC, ILT-2-APC, ILT-4-APC, and KIR2DL4-APC. These antibodies were purchased from Biolegend (San Diego, CA, USA). IL-8-APC, IP-10-APC, VEGF-APC, placental growth factor (PLGF)-APC, and interferon (IFN)-γ-APC were purchased from BD Biosciences (San Jose, CA, USA). Homologous IgG antibodies conjugated with the same fluorescent dye were used as negative controls. Approximately 1–3 × 10^6^ decidua mononuclear cells were resuspended in 100 µl PBS containing 5% NaN_3_ and 5% FBS. The antibodies targeting specific surface markers for NK cell staining was performed according to the manufacturer’s protocol and incubated in the dark for 30 min at 4°C. After fixation and permeabilization with Fixation and Permeabilization solution (BD Biosciences, San Jose, CA, USA) for 30 min, cells were stained with antibodies targeting specific intracellular cytokines at 4°C for 30 min. The cells were resuspended in 300 µl PBS containing 5% FBS, and then, 100,000 cells per sample were collected for analysis using a standard FACS Calibur flow cytometer (BD Biosciences, San Jose, CA, USA) and analyzed using the FlowJo software (TreeStar, San Carlos, CA, USA).

### Real-time PCR

Real-time PCR was used to measure the mRNA levels of interleukin (IL)-8, IFN-inducible protein (IP)-10, VEGF, PLGF, and IFN-γ in decidual tissues. Total RNA was extracted from 1 cm^3^ decidual tissue or 1 × 10^6^ dNK cells using the Trizol reagent (Invitrogen, Carlsbad, CA, USA). The cDNA was synthesized from 500 ng of total RNA using PrimeScript RT Master Mix in a total volume of 10 µl at 37°C for 15 min, 85°C for 5 s (TaKaRa, Japan) and 4°C for storage. Next, real-time PCR was run based on the detected fluorescence with SYBR Premix Ex Taq (TaKaRa). The GAPDH mRNA levels were used in the mRNA levels of the standard. PCR was performed using specific primers (Table 1). The PCR reaction involved preliminary denaturation at 95°C for 3 min, followed by 40 cycles of denaturation at 95°C for 5 s, annealing at 60°C for 30 s, and elongation at 72°C for 10 s. The RNA samples were assessed for purity and concentration using spectrophotometry (Nanodrop-2000, Thermo Scientific).

**Table 1:** Details of primer sequences for real-time PCR.

Primer set	Sequence (5′–3′)
GAPDH	F:GCACCGTCAAGGCTGAGAAC
	R:TGGTGAAGACGCCAGTGGA
Interleukin-8	F:TTTCAGAGACAGCAGAGCACACAA
	R:CACACAGAGCTGCAGAAATCAGG
IP-10	F:GGCCATCAAGAATTTACTGAAAGCA
	R:TCTGTGTGGTCCATCCTTGGAA
Vascular endothelial growth factor	F:GAGCCTTGCCTTGCTGCTCTA
	R:CACCAGGGTCTCGATTGGATG
Placental growth factor	F:GAGACGGCCAATGTCACCA
	R:GCTGAGAGAACGTCAGCTCCA
Interferon-γ	F:CTTTAAAGATGACCAGAGCATCCAA
	R:GGCGACAGTTCAGCCATCAC

### Multiplex Cytokine Assays

The secretory cytokines of the decidual tissues and dNK cells were detected using a multiplex cytokine assay (Magpix, Bio-Rad Laboratories, Veenendaal, The Netherlands) according to the manufacturer’s instructions. The Human Magnetic Luminex Screening 5 Plex Assay kit (R&D systems, Minneapolis, MN, USA) contained beads conjugated with monoclonal antibodies for IL-8, IP-10, VEGF, PLGF, and IFN-γ. Cytokine assays sensitivity and measurement ranges have shown in Table S1 in Supplementary Material.

### Matrigel Invasion Assay

HTR-8/SVneo is the human first-trimester trophoblast cell line (from University of Toronto, ON, Canada) and has the similar function to EVTs. We determined the ability of dNK cells to induce the invasion of HTR-8/SVneo cells *in vitro* by Transwell (Millipore, Billerica, MA, USA) assays. First, 200 µl of HTR-8/SVneo cells at a concentration of 5 × 10^5^ cells/ml were plated in invasion chambers, which were immersed in 24-well cell culture plates containing 500 µl RPMI 1640 with 10% FBS (control) or the supernatant of dNK cells from the IA or RSA samples. The plates were cultured at 37°C and 5% CO_2_. After 24 h, using cotton swabs, non-invading cells were removed from the top of the Matrigel (BD Biosciences, San Jose, CA, USA). The cells that had invaded the Matrigel were fixed using 4% paraformaldehyde and dyed using 0.1% crystal violet. Non-overlapping fields at 100× magnification were analyzed using a light microscope.

### Tube Formation Assay

The human umbilical vein endothelial cells (HUVECs) are isolated from pooled donors. Experiments on HUVECs were carried out at the 4–6 passages. The ability of HUVECs to form network-like structures *in vitro* was examined. 25 µl of HUVECs at a concentration of 1.2 × 10^6^ cells/ml was placed in 96-well plate pre-coated with 50 µl/well Matrigel (BD Biosciences, Bedford, MA, USA), and 25 µl of supernatants of dNK cells from the two groups were added to 96-well plate and incubated at 37°C, 5% CO_2_. The HUVECs were photographed microscopically to evaluate the extent of tube formation after 6 h. Non-overlapping fields at 40× magnification were analyzed using a light microscope.

### HTR-8/SVneo Cell Line Culture and Transfection

HTR-8/SVneo cells were cultured in RPMI 1640 medium containing 10% FBS. The transfection methods were used as described previously (30). Briefly, 1 × 10^6^ HTR-8/SVneo cells were plated in 6-well plates and transfected with miR-133a mimics, inhibitor, or negative control using Lipofectamine 2000 (Invitrogen, Carlsbad, CA, USA) according to the manufacturer’s instructions, when the cells reached 50–60% confluence.

### Western Blot Analysis

The methods of western blot analysis were described previously (28). Briefly, HTR-8/SVneo cells transfected with miRNAs were washed by PBS and lysed by RIPA lysis buffer. Samples consisting of 50 µg total protein were loaded onto an SDS-PAGE gel (P0012AC, Beyotime Biotechnology, China) and transferred electrophoretically to nitrocellulose membranes (LC2000, Invitrogen). After blocking with Tris-buffered saline with Tween-20 (TBST) containing 5% milk powder, the membranes were incubated with the appropriate primary antibody against HLA-G (MEM-G/1, ab7759; Abcam, Cambridge, UK, 1:500) or tubulin (ab6161, Abcam, Cambridge, UK, 1:2,000), anti-mouse IgG horseradish peroxidase (HRP)-conjugated secondary antibody (GE Healthcare, Piscataway, NJ, USA) was added for 1 h.

The blots were developed using Immobilon Western HRP Substrate Luminol Reagent (Millipore, Billerica, MA, USA). The quantification of HLA-G relative to tubulin expression within each sample was determined using the QuantiOne imaging software (Bio-Rad, USA).

### Measurement of Soluble HLA-G Production in Culture Supernatants

The methodology to measure HLA-G using ELISA was described previously. Culture supernatants of HTR-8/SVneo cells transfected with miR-133a were harvested. Soluble HLA-G was quantified using MEM-G/9 (ab7758, Abcam, Cambridge, UK), which recognizes the most abundant soluble isoforms and anti-human β2-microglobulin as capture and detection antibodies, respectively (31). The plates were incubated for 30 min with substrate and absorbance was measured at 450 nm using a Benchmark microplate reader (Bio-Rad, USA).

### Cocultures of dNK Cells and HTR-8/SVneo

The coculture system of fresh isolated CD56^+^CD94^+^ dNK cells (5 × 10^5^) of IA patients and HTR-8/SVneo cells (5 × 10^5^) transfected with miR-133a relative sequence (1:1 ratio) was established and then seeded onto 24-well culture plates in 1 ml RPMI 1640 medium containing 10% FBS without IL-15. In addition, the fresh isolated dNK cells of IA patients with HTR-8/SVneo cells in RPMI 1640 medium containing 10% FBS plus blocking antibodies for KIR2DL4 (clone mab33) or isotype controls at a concentration of 10 µg/ml were cocultured.

### Statistical Analysis

The data from independent experiments were presented as the mean ± SD. Differences between two groups were analyzed by Student’s *t*-test, and multiple groups were analyzed by the one-way analysis of variance. A *P*-value < 0.05 was considered to be statistically significant. All analyses were performed using SPSS 19.0 software (SPSS, Chicago, IL, USA).

## Results

### Identification of Human Normal and Abnormal First-Trimester dNK Cells

**Figure 1:**
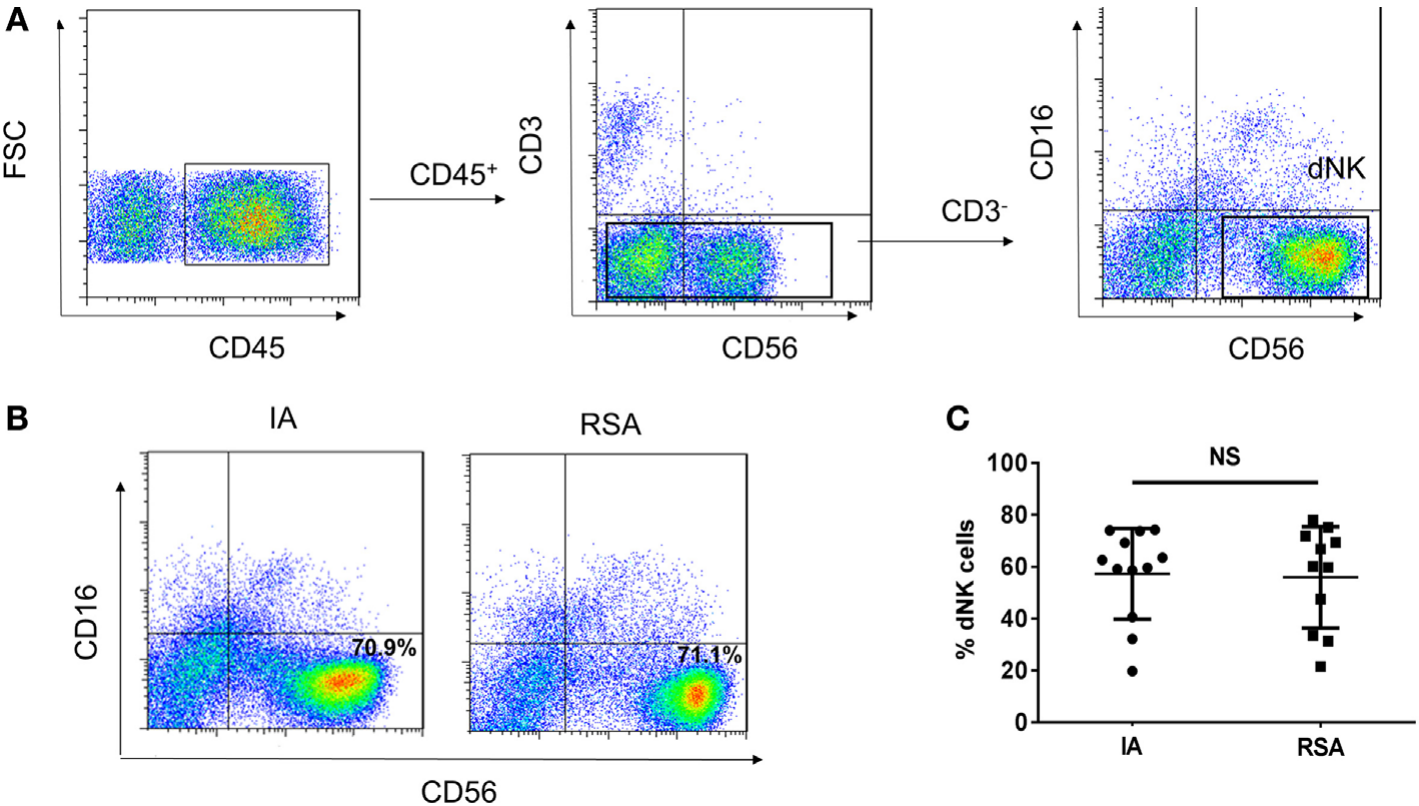
CD56^bright^CD16^−^ natural killer (NK) cells present in large numbers in human deciduas of induced abortion (IA) group and recurrent spontaneous abortion (RSA) group. **(A)** Representative density plots showing the CD56^bright^ NK (dNK) cells in gated CD45^+^CD3^−^CD56^+^CD16^−^ NK cells isolated from decidua in the first trimester of IA group and RSA group. **(B)** Representative density plots showing the decidual NK (dNK) cell phenotypes of the two groups. **(C)** Percentages of dNK cells in gated decidual lymphocytes. *n* = 12 and 11 for IA and RSA, respectively. The data in B are presented as the means ± SD. Student’s *t*-test.

To characterize the phenotypes of dNK cells in patients with IA and patients with RSAs, flow cytometry analysis was performed with CD45, CD3, CD56, and CD16 mAbs. To exclude the confounding fluorescent signals from other cells, only CD45^+^ leukocytes were examined, and CD3^−^CD56^bright^CD16^−^ NK cells were regarded as dNK cells (Figure 1A). In both IA and RSA samples, the CD56^bright^CD16^−^ NK cells were the dominant lympholeukocyte cell type in the decidua (~70%) (Figure 1B). The percentage of dNK cells exhibited no significant difference between the groups (*P* > 0.05, Figure 1C).

### Lower KIR2DL4 Expression in dNK Cells in the RSA Group than in the IA Group

**Figure 2:**
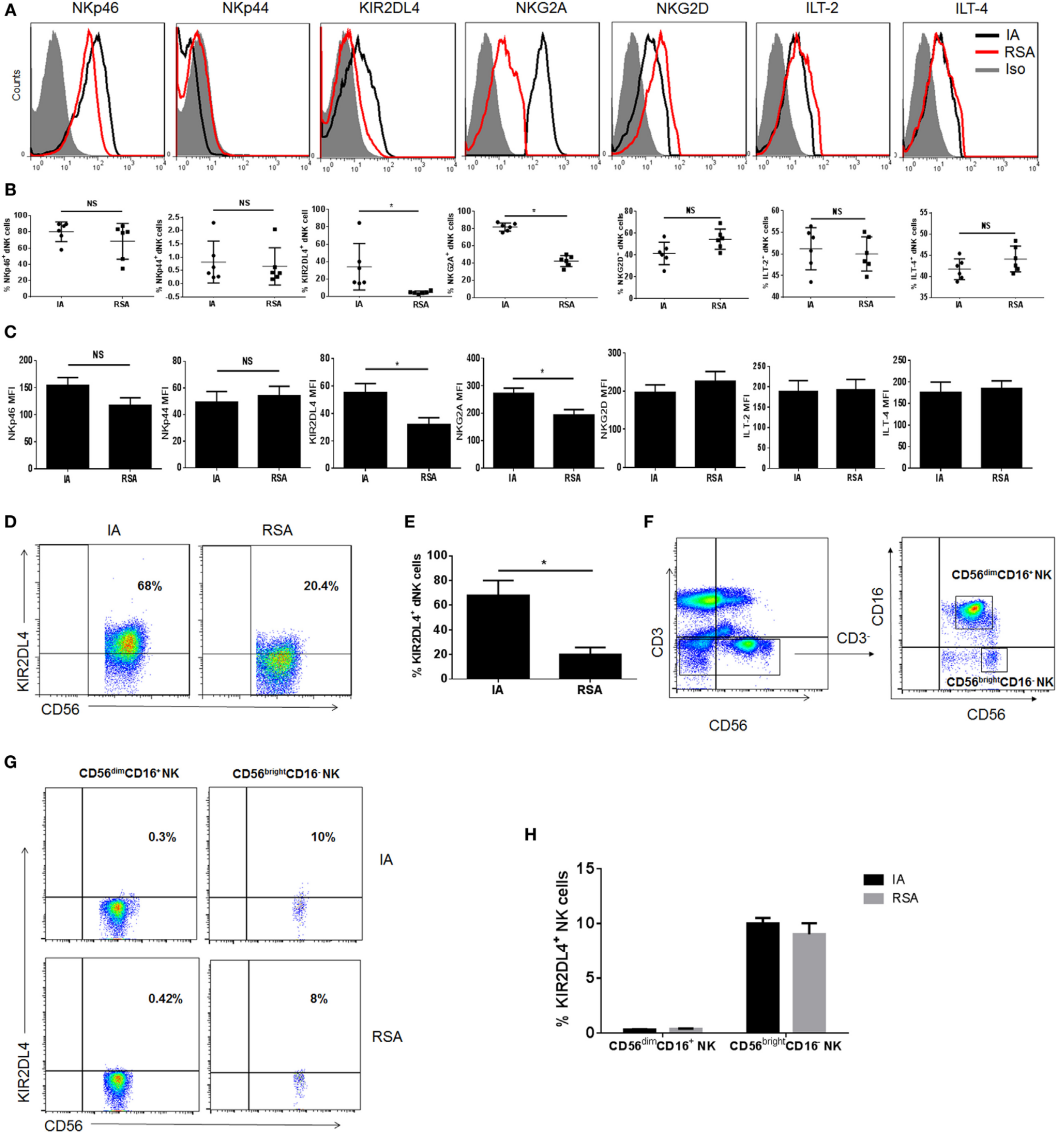
Expression of natural killer (NK) cell receptors in decidual NK (dNK) cells of induced abortion (IA) and recurrent spontaneous abortion (RSA). **(A)** Expressions of NKp46, NKp44, killer immunoglobulin-like receptor 2DL4 (KIR2DL4), NKG2A, NKG2D, immunoglobulin-like transcript 2 (ILT2), and ILT4 on the CD3^−^CD56^+^CD16^−^ dNK cells of the two groups. The gray-filled histograms represent background staining of corresponding isotype-matched control. **(B)** Percentage of NKp46, NKp44, KIR2DL4, NKG2A, NKG2D, ILT2, and ILT4 on CD3^−^CD56^+^CD16^−^ dNK cells of IA and RSA. **(C)** Mean fluorescence intensity (MFI) of these receptors on CD3^−^CD56^bright^CD16^−^ dNK cells of IA and RSA. **(D)** Representative density plots showing the KIR2DL4 expression on gated CD3^−^CD56^bright^CD16^−^ dNK cells of IA and RSA with intracellular staining. **(E)** Percentage of KIR2DL4 expression on gated CD3^−^CD56^bright^CD16^−^ dNK cells of two groups with intracellular staining. **(F)** Representative density plots showing CD56^bright^ and CD56^dim^ NK cells in gated CD56^+^CD3^−^ NK cells isolated from peripheral blood (pNK) in the first trimester of IA group. **(G)** Representative density plots showing the KIR2DL4 expression on gated CD3^−^CD56^bright^ and CD3^−^CD56^dim^ pNK cells of IA and RSA. **(H)** Percentage of KIR2DL4 expression on gated CD3^−^CD56^bright^ and CD3^−^CD56^dim^ pNK cells of IA and RSA. *n* = 6 for each group. The data are presented as the means ± SD. *P < 0.01 Student’s *t*-test.

Cell surface receptors, including NKp44, NKp46, NKG2D, NKG2A, ILT2, ILT4, and KIR2DL4 on dNK cells in both groups, were subsequently investigated by flow cytometry. Among these receptors, ILT2, ILT4, and KIR2DL4 were the ligands of HLA-G. Six samples of the RSA group and six samples of the IA group were measured. We found that the expression of NKp44, NKp46, NKG2D, ILT2, and ILT4 on dNK cells showed no significant differences between the groups (*P* > 0.05, Figures 2A–C). However, the RSA group expressed lower KIR2DL4 and NKG2A than did the IA group in terms of both the percentage and mean fluorescence intensity (*P* < 0.05, Figures 2A–C). Considering KIR2DL4 resides predominantly in endosomes, we examined the KIR2DL4 expression with intracellular staining and found that the RSA group also expressed lower KIR2DL4 in endosomes (*P* < 0.05, Figures 2D,E). Additionally, KIR2DL4 expression on CD56^dim^ and CD56^bright^ peripheral blood NK (pNK) cells were detected both in the IA and the RSA group. The results demonstrated that CD56^dim^ pNK cells scarcely expressed KIR2DL4, while CD56^bright^ pNK cells have low expression of KIR2DL4 in both two groups (*P* > 0.05, Figures 2F–H).

### Differences in Secretion Functions of dNK Cells in the RSA and IA Groups

**Figure 3:**
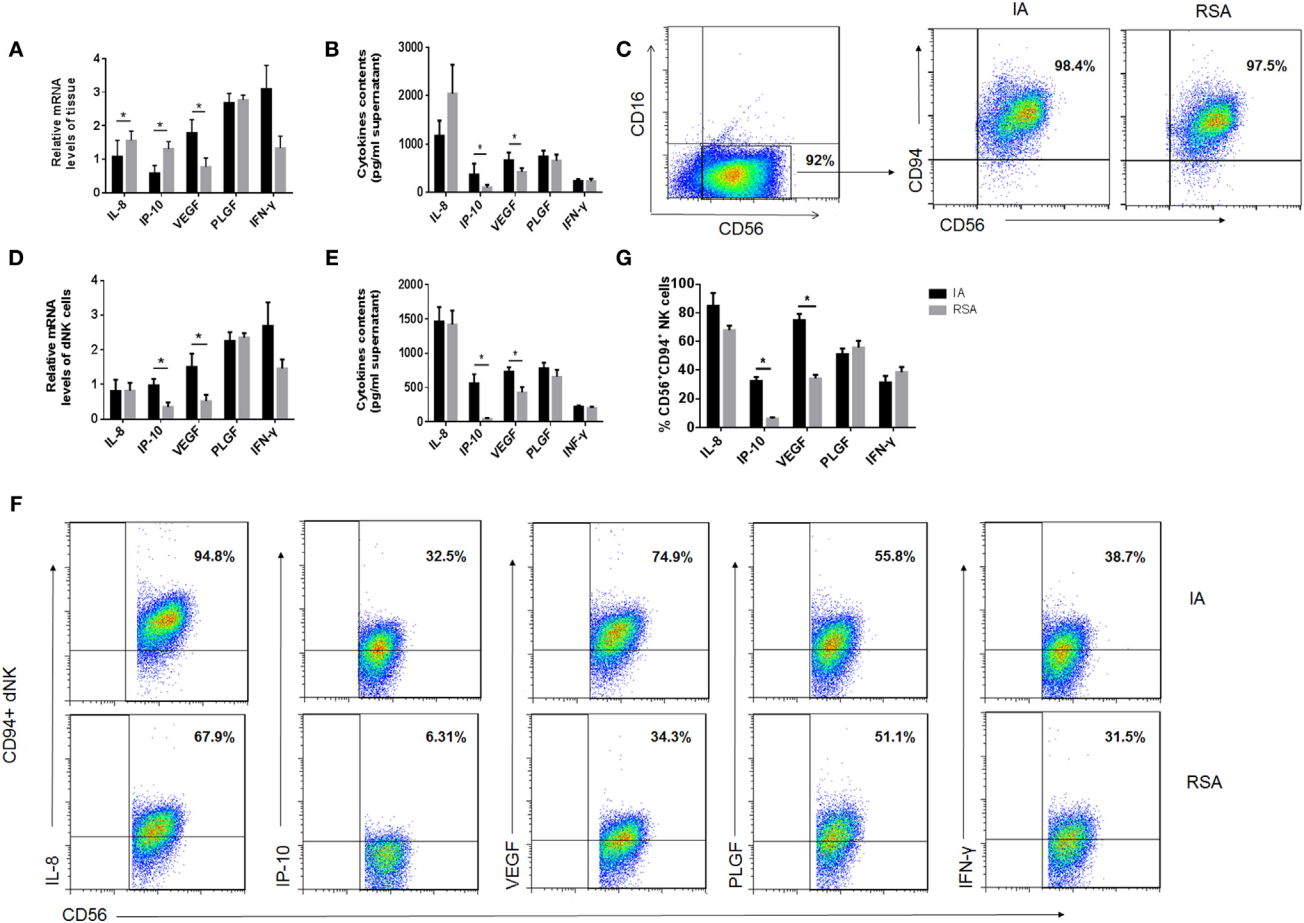
Cytokine production by human decidual NK (dNK) cells of the two groups. **(A)** mRNA levels and **(B)** protein levels of cytokines in decidual tissues of the two groups. **(C)** Representative purity analysis of sorted CD56^+^CD16^−^ natural killer (NK) cells and CD56^+^CD94^+^ NK cells in induced abortion (IA) and recurrent spontaneous abortion (RSA). The purified CD56^+^CD94^+^ NK cells were subsequently used for real-time PCR and coculture. **(D)** mRNA levels of cytokines in fresh purified CD56^+^CD94^+^ dNK cells and **(E)** protein levels of cytokines in CD56^+^CD94^+^ dNK cultured 24 h after purification. **(F)** Representative density plots showing the cytokines in CD56^+^CD94^+^ dNK cells of IA and RSA with intracellular staining. **(G)** Percentage of cytokine expression in CD56^+^CD94^+^ dNK cells of IA and RSA, *n* = 6 for each group. The data are presented as the means ± SD. *P < 0.05 Student’s *t*-test.

Owing to the ability of dNK cells to secrete cytokines that may affect pregnancy, the profile of the cytokine secretion of dNK cells in the two groups was examined using a multiplex cytokine assay. The CD56^bright^ dNK cells were purified with immunomagnetic beads, and CD56^+^CD94^+^ dNK cells were further purified by FCM and more than 97% of dNK were CD94^+^CD56^+^CD16^−^ NK cells in two groups (Figure 3C). We first examined the mRNA levels of certain cytokines, including IL-8, IP-10, VEGF, PLGF, and IFN-γ, in the decidual tissues and dNK cells in each group. We found that the IL-8 and IP-10 levels were higher in the decidual tissues of the RSA group than in the IA group but that the VEGF level in RSA was lower than that in IA (*P* < 0.05, Figure 3A). However, the mRNA levels of IP-10 and VEGF in CD56^+^CD94^+^ dNK cells were much lower in the RSA group (*P* < 0.05, Figure 3D). Then, the supernatants of decidual tissue homogenates and the supernatants of CD56^+^CD94^+^ dNK cells, which were cultured for 24 h, were harvested and used for the analysis of these cytokines. All CD56^+^CD94^+^ dNK cell cultures were supplemented with 20 ng/ml IL-15. We found significantly higher levels of IP-10 and VEGF in both tissue homogenate supernatants and dNK cell supernatants in the IA group (*P* < 0.05, Figures 3B,E). Meanwhile, all these cytokines in CD94^+^CD56^+^CD16^−^ NK cells were detected by intracellular staining and analyzed by FCM. The results also demonstrated that IP-10^+^ CD56^+^ dNK cells and VEGF^+^ CD56^+^ dNK cells were significantly lower in the RSA group than the IA group (*P* < 0.05, Figures 3F,G).

### Difference in the Proinvasive and Proangiogenesis Ability of dNK Cells in the RSA and IA Groups

**Figure 4:**
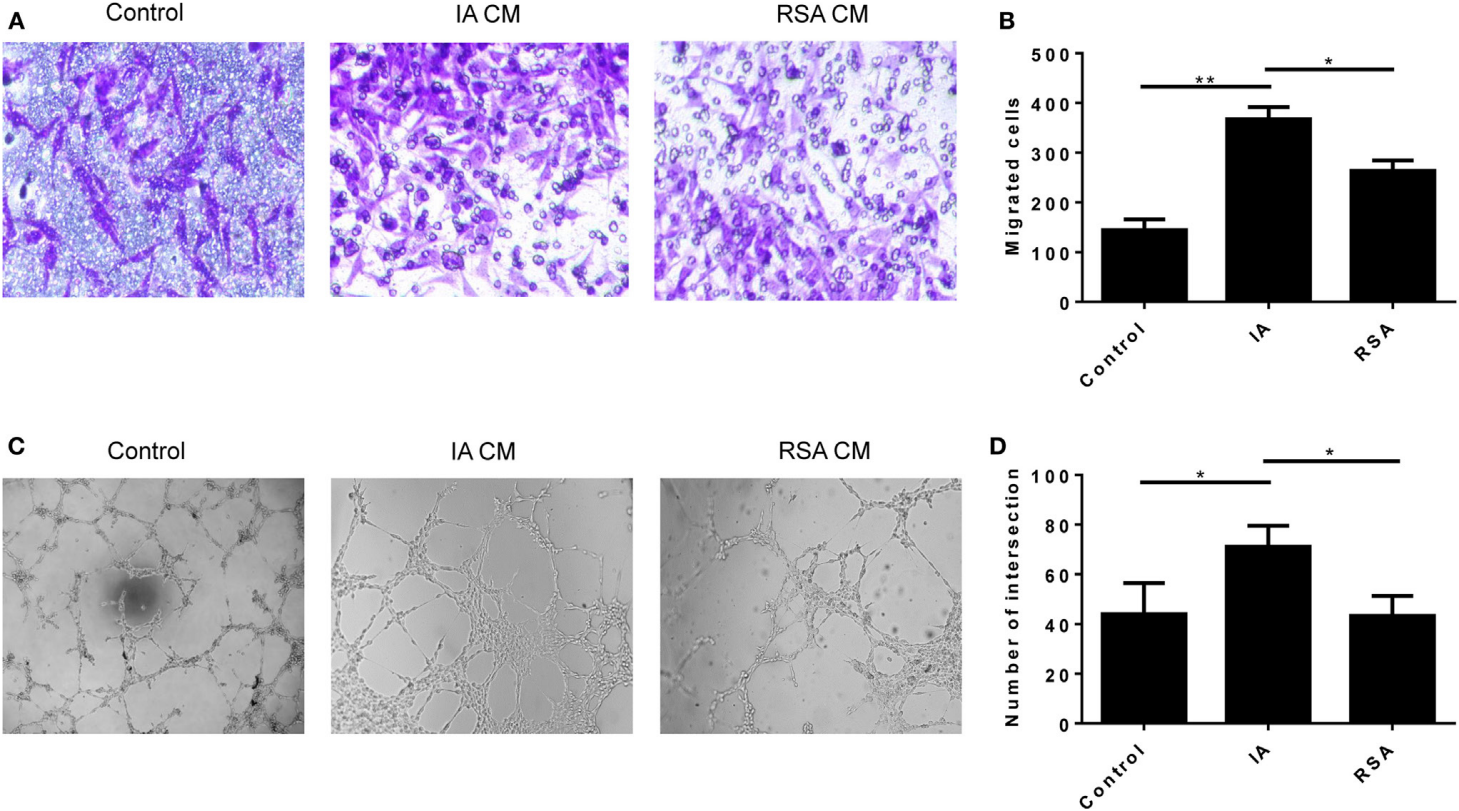
Decidual NK (dNK) cells of recurrent spontaneous abortion (RSA) reduce HTR-8/SVneo invasion and human umbilical vein endothelial cell (HUVEC) tube formation. **(A,B)** Transwell assay showed CD56^+^CD94^+^dNK cells culture supernatants of the two groups affects the number of invading HTR-8/SVneo cells. **(A)** Representative images of invading HTR-8/SVneo cells which were dyed with crystal violet. Magnification, 100×. **(B)** Average number of HTR-8/SVneo cells invading across the Matrigel toward dNK cells of different groups. **(C,D)** Formation by HUVECs of tube structures *in vitro* in conditioned supernatants obtained from CD56^+^CD94^+^ dNK cells of different groups. Values shown on each frame indicate average number of intersection. Magnification, 40×. The data are representative of three experiments. The data in panels **(B,D)** are presented as the means ± SD. **P < 0.01 versus control and induced abortion (IA) group. *P < 0.05 versus IA and RSA group. Student’s *t*-test.

To examine whether the variation of cytokines changed the proinvasive ability of dNK cells, we conducted the Transwell assay. The HUVEC tube formation assay was done to test the proangiogenesis property of dNK cells. The CD56^+^CD94^+^ dNK culture supernatants of the two groups were harvested and cocultured with HTR-8/SVneo cells to perform the Matrigel invasion assay. At the same time, the supernatants were cocultured with HUVECs to perform the tube formation assay. We confirmed that the CD56^+^CD94^+^ dNK supernatants of the RSA group significantly reduced the invasive activity of HTR-8/SVneo cells compared with the IA group (*P* < 0.05, Figures 4A,B). The results of the tube formation assay also revealed that CD56^+^CD94^+^ dNK supernatants of the RSA group significantly reduced the tube formation ability of HUVECs (*P* < 0.05, Figures 4C,D). All dNK cells were cultured with 20 ng/ml IL-15 for 24 h before harvesting the supernatants.

### Decreased HLA-G Expression by miR-133a Affected dNK Function

**Figure 5:**
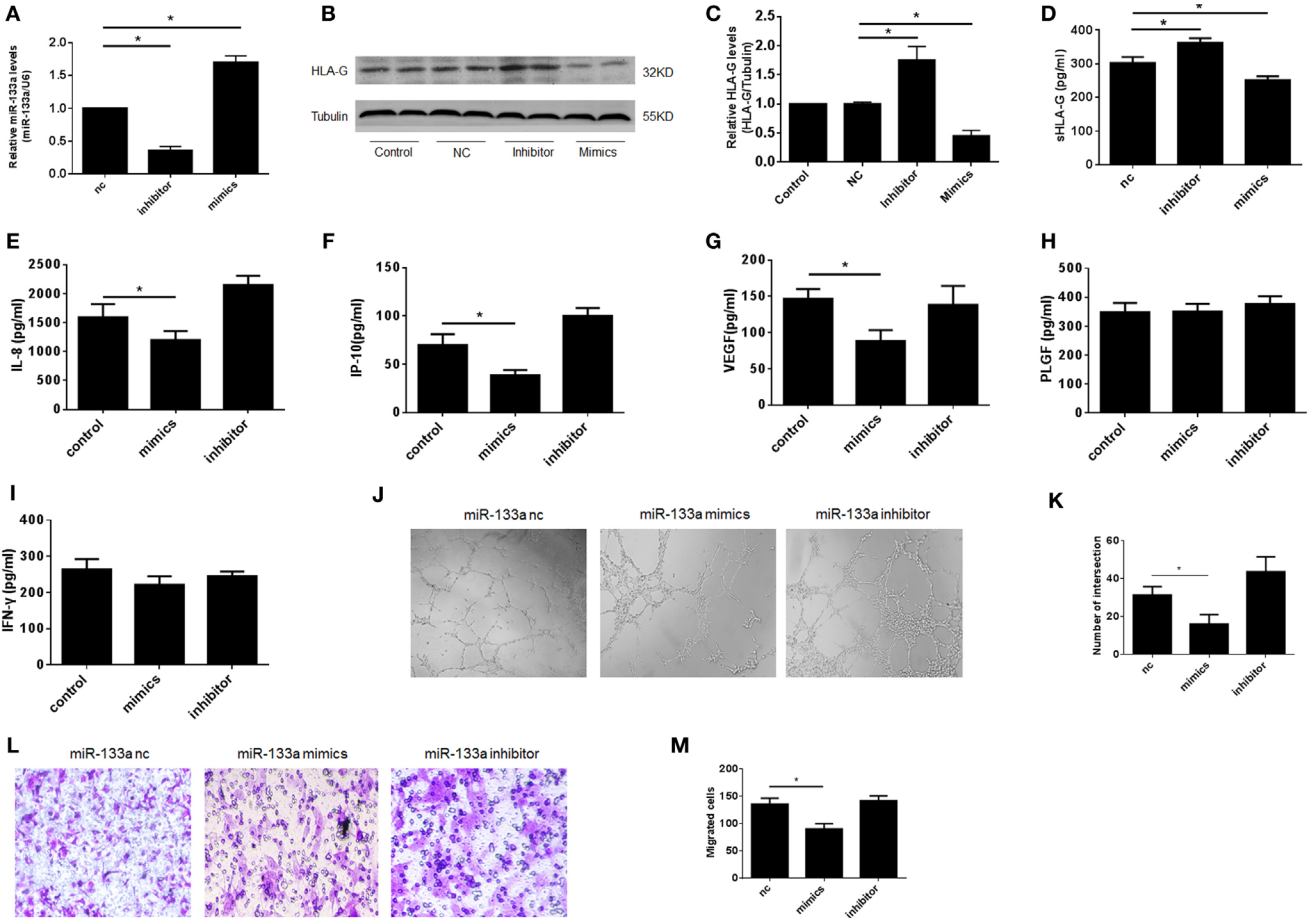
Decreased human leukocyte antigen (HLA)-G expression by miR-133a affected the function of decidual NK (dNK), including proinvasion and proangiogenesis. Overexpression of miR-133a reduced HLA-G expression in HTR-8/SVneo cells. **(A)** Relative miR-133a expression after transfection with mimics, inhibitor, and negative control (nc) in HTR-8/SVneo cells by qRT-PCR. **(B,C)** Relative HLA-G protein levels after transfection with miR-133a by western blot. The data are representative of three experiments. **(D)** Soluble HLA-G concentration in supernatant of HTR-8/SVneo cells transfected with miR-133a for 24 h. Cell culture supernatants were analyzed for **(E)** interleukin (IL)-8, **(F)** IP-10, **(G)** vascular endothelial growth factor (VEGF), **(H)** placental growth factor (PLGF), and **(I)** INF-γ.**(J,K)** Tube formation by human umbilical vein endothelial cells *in vitro* in conditioned supernatants obtained from CD56^+^CD94^+^ dNK cells cocultured with HTR-8/SVneo transfected with miR-133a negative control (nc), inhibitor, or mimics. Magnification, 40×. **(L,M)** Number of invading HTR-8/SVneo cells in conditioned supernatants obtained from CD56^+^CD94^+^ dNK cocultured with HTR-8/SVneo transfected with miR-133a nc, inhibitor, or mimics. Magnification, 100×. The data are representative of three experiments. The data are presented as the means ± SD. *P < 0.05. Student’s *t*-test.

In our previous study, we found that miR-133a was greatly overexpressed in RSA villi compared to those from IA patients, and we confirmed that miR-133a was most likely to bind to the HLA-G 3′UTR (28). To examine whether the downregulation of HLA-G by miR-133a affected the function of dNK cells, we prepared a coculture system involving HTR-8/SVneo cells transfected with miR-133a and dNK cells isolated from IA patients. We first identified the efficiency of the miR-133a transfection with real-time PCR (Figure 5A). Next, we confirmed the downregulation of HLA-G by miR-133a using western blot (Figures 5B,C). The culture supernatants were harvested 24 h later after transfection and sHLA-G concentrations were detected by ELISA. We confirmed that sHLA-G could also be downregulated by miR-133a (Figure 5D). To investigate the role of HLA-G in controlling dNK secretion functions, fresh CD56^+^CD94^+^ dNK cells were cocultured with HTR-8/SVneo cells transfected with miR-133a. The cell culture medium was harvested, and the cytokines were analyzed (Figures 5E–I). The results of the assay showed that CD56^+^CD94^+^ dNK cells cocultured with HTR-8/SVneo cells transfected with miR-133a mimics significantly decreased the levels of IL-8, IP-10, and VEGF (*P* < 0.05, Figures 5E–G). The tube formation assay revealed that the supernatants of the CD56^+^CD94^+^ dNK cells cocultured with HTR-8/SVneo cells transfected with miR-133a mimics significantly reduced the tube formation ability of HUVECs (*P* < 0.05, Figures 5J,K). The Transwell assay also showed that the CD56^+^CD94^+^ dNK cells cocultured with HTR-8/SVneo cells transfected with miR-133a mimics significantly reduced the migration ability of the HTR-8/SVneo cells (*P* < 0.05, Figures 5L,M).

### KIR2DL4 Was Involved in the Impairment of dNK Cell Function

**Figure 6:**
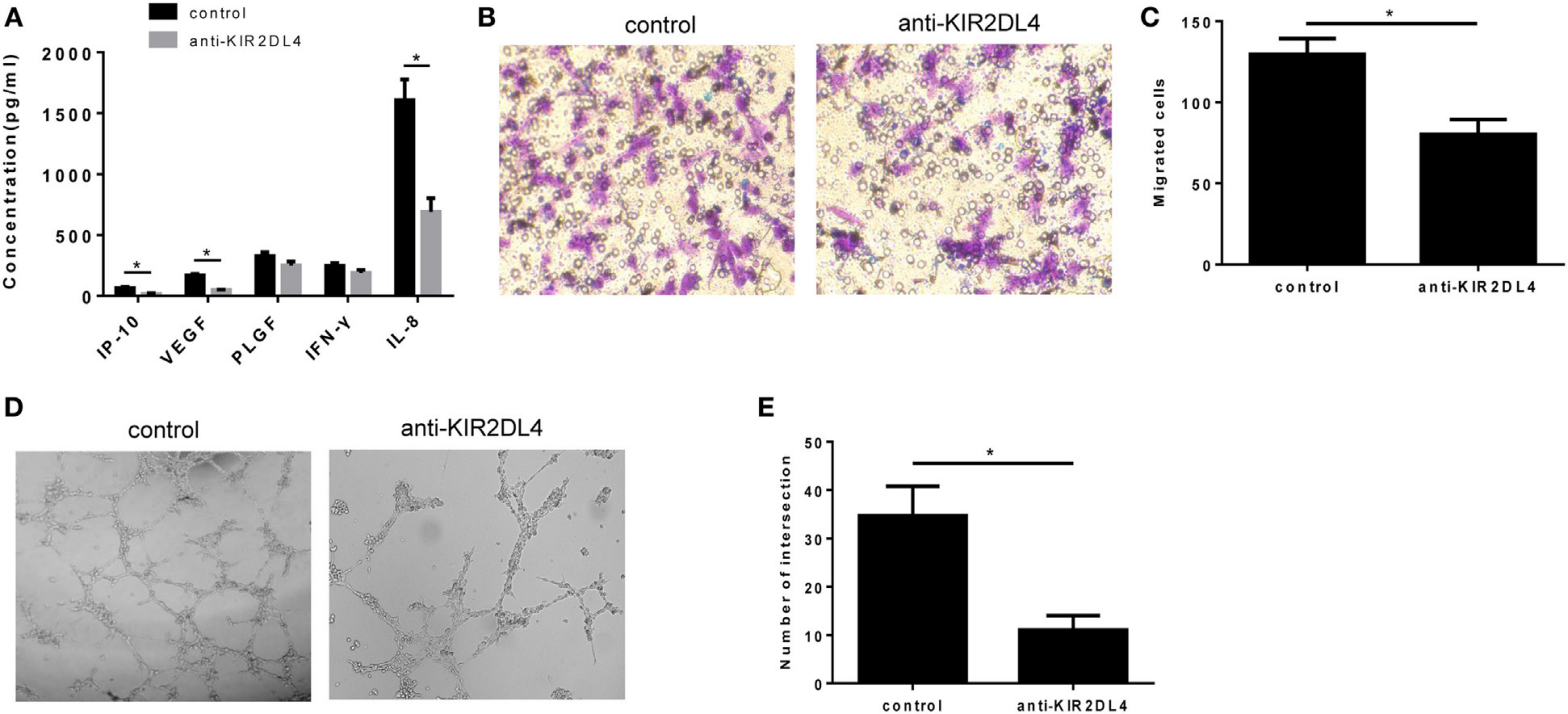
The impairment of proinvasion and proangiogenesis function of decidual NK (dNK) after blocking killer immunoglobulin-like receptor 2DL4 (KIR2DL4). **(A)** Cell culture supernatants were analyzed for IP-10, vascular endothelial growth factor (VEGF), placental growth factor (PLGF), INF-γ, and interleukin (IL)-8. The data are representative of three experiments. **(B,C)** Number of invading HTR-8/SVneo cells in conditioned supernatants obtained from CD56^+^CD94^+^ dNK cells cocultured with HTR-8/SVneo cells in the presence of anti-KIR2DL4 Ab or isotype. **(D,E)** Formation by human umbilical vein endothelial cells of tube structures *in vitro* in conditioned supernatants obtained from CD56^+^CD94^+^ dNK cells cocultured with HTR-8/SVneo in the presence of anti-KIR2DL4 antibody or isotype. The data in panels **(C,E)** are presented as the means ± SD. *P < 0.05. Student’s *t*-test.

To examine whether KIR2DL4 was involved in the impairment dNK cell function, we cocultured CD56^+^CD94^+^ dNK cells with HTR-8/SVneo cells plus anti-KIR2DL4 antibodies or isotypes controls at a concentration of 10 µg/ml. The results showed reduced IL-8, IP-10, and VEGF in the presence of blocking antibodies for KIR2DL4 (*P* < 0.01, Figure 6A). We found that the anti-KIR2DL4 antibodies reduced the invasive activity of HTR-8/SVneo cells compared with the control group (*P* < 0.05, Figures 6B,C). In addition, tube formation by HUVECs was observed after 6 h with supernatant stimulation. The results showed the significantly reduced tube formation capacity following the blockade of KIR2DL4 by Ab (*P* < 0.05, Figures 6D,E).

## Discussion

During pregnancy, there is complicated immunological regulation at the maternal–fetal interface that is necessary for a successful pregnancy. The dNK cells are the major component of the immune cells present in the decidua between HLA-G^+^ extravillous cytotrophoblasts that are essential for early pregnancy. Increasing research has suggested that HLA-G and dNK cells have unique features and play important roles at the maternal–fetal interface. However, the specific mechanisms are still unknown.

Our present study demonstrated that decreased HLA-G expression by miR-133a transfection played an important role in the regulation of the secretory functions of dNK cells. We found that there were no significant differences between RSA and IA patients regarding the percentage of dNK cells in decidual lymphocytes. However, regarding to the receptors of dNK cells, especially KIR2DL4, the expression level was lower in RSA than in IA. This finding was consistent with a previous report (32). Although KIR2DL4 has a long cytoplasmic domain and contains ITIMs, which can transduce inhibitory signals to NK cells, it could enhance dNK activity of cytokine and chemokine secretion when bound to HLA-G. KIR2DL4 could also activate endosomal signaling for a proinflammatory or proangiogenic response (22). To date, why the dNK cells of RSA patients have lower KIR2DL4 is still unknown. We speculated that hematological and immunological homeostasis changed the uterine microenvironment so as to disturb the differentiation of dNK cells in RSA patients.

As we know, dNK cells are CD56^bright^CD16^−^ NK cells which overexpress CD94 have strong secretory functions. Considering the human decidua contain group 3 ILC which express CD56 and NCR but lack CD94/NKG2A and KIR, and these NCR^+^ ILC3 cells could also produce IL-8. We separated CD56^+^CD94^+^ dNK cells by flow cytometry for excluding ILC3 cells. We confirmed that CD94^+^CD56^+^ NK cells could secret certain cytokines, such as IL-8, IP-10, and VEGF, etc. Thus, we examined whether the decreased expression of KIR2DL4 could influence dNK cells’ secretion capability. We found that the mRNA levels of IL-8 and IP-10 were increased, whereas the VEGF levels were decreased in the decidual tissues of the RSA group. However, the protein levels of IL-8 and VEGF were in accordance with the mRNA levels in the decidual tissues from the RSA group, whereas the protein levels of IP-10 were not the same as the mRNA levels. In addition, IP-10 and VEGF were decreased in dNK cells of RSA at both the mRNA and protein levels. Although IL-8 seemed to be increased in dNK cells of RSA, there was no significant difference. IL-8 is a chemokine that is produced by decidual cells and dNK cells (33) and can bind to two receptors, IL-8RA (CXCR1) and IL-8RB (CXCR2) (34). Both IL-8RA and IL-8RB were detected on the EVTs (35). IL-8 may lead to the release of matrix metalloproteinase-2 and MMP-9, thereby increasing the EVT and HTR-8/SVneo cell invasion (35, 36). Decreased expression of IL-8 is associated with RSA (37). Our study demonstrated that IL-8 was increased in decidual tissues but was reduced in dNK cells, so we thought that IL-8 was produced not only by dNK cells but by other decidual cells. The higher expression of IL-8 in decidual tissues might have been due to other decidual cells’ compensatory mechanisms. However, our conclusion still needs to be further studied due to the limited sample size of our study. IP-10 is also one of the chemokines secreted by endometrial stromal and glandular cells (38). IP-10 can promote trophoblast invasion, but its mechanism is still unknown. We considered the supernatants of cultured dNK cells from the RSA group had weakened the invasion ability of HTR-8/SVneo cells compared with the IA group due to the decreased levels of IL-8 and IP-10, which were both proinvasion cytokines. VEGF is a strong angiogenic factor that is secreted by the decidua and was proven to be secreted by dNK cells (15, 39). VEGF is crucial for vascular growth, which is one of the processes for placental development (40). Lower VEGF expression at the maternal–fetal interface was reported to be related to RSA (41). VEGF and VEGF soluble receptor-1 (sFlt-1) in serum were both increased in RSA because of placental ischemia/hypoxia and endothelial dysfunction (42). However, another study showed that reduced serum VEGF may contribute to RSA (43). Our results demonstrated that VEGF was decreased in both the decidual tissues and dNK cells of the RSA group, which may affect the tube formation of HUVECs. In our studies, the decreased expression of VEGF was related to a lower tube formation ability of HUVECs.

Numerous studies have indicated that HLA-G^+^ extravillous cytotrophoblasts contacted by dNK cells play a crucial role in the maintenance of pregnancy (6, 44, 45). KIR2DL4, as a particular receptor for HLA-G on dNK, may play a mediation role in modulating the secretion ability of dNK cells. Our previous study found that miR-133a was highly expressed in the villi of the RSA group (28). Multi-software prediction and real-time PCR confirmed that miR-133a could bind to the 3′UTR of HLA-G (28). It was also confirmed that HLA-G (all HLA-G isoform and soluble HLA-G) was downregulated by miR-133a in HTR-8/SVneo cells. The dNK cells of IA patients, when cocultured with HTR-8/SVneo cells transfected with miR-133a mimics, decreased the expression of IL-8, IP-10, and VEGF. These cytokines in the coculture supernatants plus the anti-KIR2DL4 antibody were also significantly decreased. The results suggested that low levels of HLA-G or KIR2DL4 may influence the secretion of dNK cells in a way that affected the invasion capability of HTR-8/SVneo cells and tube formation of HUVECs. The mechanism of HLA-G influencing the secretion of dNK cells was reported that soluble HLA-G stimulate resting NK cells on endocytosis into endosomes and combined with KIR2DL4 which stimulated senescent NK cells to promote vascular remodeling and angiogenesis probably by secreting certain cytokines, such as IL-8 (16). KIR2DL4 is reported to be related to vascular remodeling and breast cancer invasion (16, 46). We know that vascular remodeling and trophoblast cell invasion are two key processes of early pregnancy. Trophoblast cells have invasion abilities just like tumor cells. Our study found that KIR2DL4 could also influence trophoblast cell lines’ invasion, demonstrating that KIR2DL4 on dNK is crucial for HLA-G interfaced dNK cells and the maintenance of a successful pregnancy.

**Figure 7:**
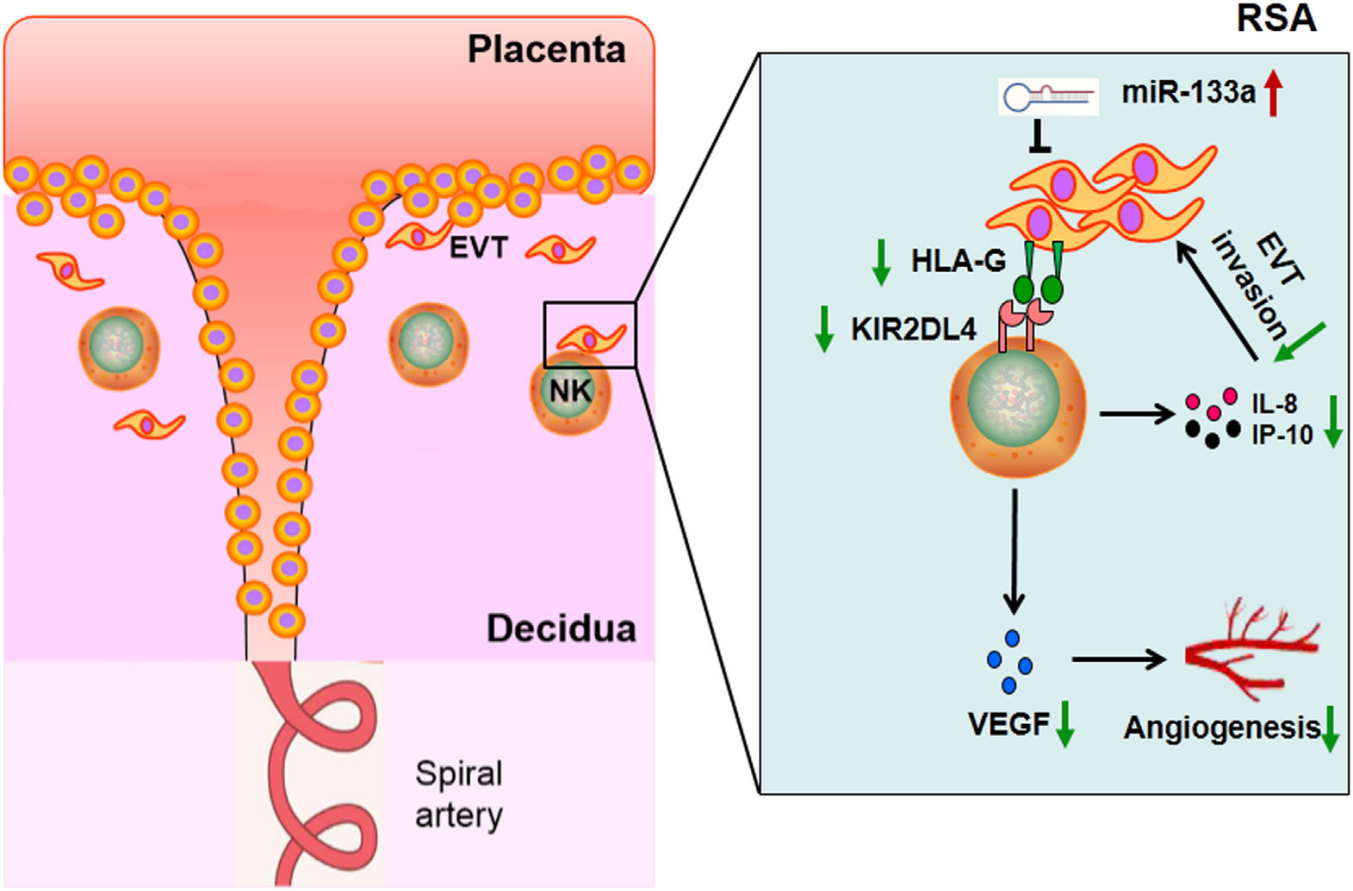
Proposed scheme depicting the hypothesis that decreased human leukocyte antigen (HLA)-G expression impaired functions of decidual NK cells in recurrent spontaneous abortion (RSA).

In conclusion, our current study showed that lower KIR2DL4 expression on dNK cells of RSA group could downregulate the proinvasion and proangiogenic cytokine secretion of dNK cells. Furthermore, it was indicated that decreased HLA-G expression by miR-133a in the trophoblast cell line, HTR-8/SVneo could influence the secretion ability of dNK cells when bound to KIR2DL4. The decreased cytokines could affect trophoblast invasion and angiogenesis (Figure 7). Our findings provide a possible mechanism of RSA and a basis for further study; in addition, this study may provide a potential drug target for therapy of RSA.

## Ethics Statement

This study was approved by the Institutional Review Board, Tang Du Hospital, Fourth Military Medical University. All subjects gave written informed consent in accordance with the Declaration of Helsinki.

## Author Contributions

The mainly task of the research, including experiment and sorting results, was completed by WG. LC and XW were guiding the research and offering the funds supporting collectively. LF contributed to the quality control of the experiment and the article writing. BL, XX, SC, and JW contributed to offer the experiment site and technical supporting selflessly. Especially, gratitude is due to FY for her assistance in specimen collection.

## Conflict of Interest Statement

The authors declare that the research was conducted in the absence of any commercial or financial relationships that could be construed as a potential conflict of interest.
