# Supplementary material for: Decreased Human Leukocyte Antigen-G Expression by miR-133a Contributes to Impairment of Proinvasion and Proangiogenesis Functions of Decidual NK Cells
Source: Front Immunol. 2017 Jun 28;8:741. doi: 10.3389/fimmu.2017.00741 (PMC5487407; doi:10.3389/fimmu.2017.00741)
Supplement: Supplementary file 1 [file table_1.docx]

Table S1. Sensitivity and measurement ranges in cytokine assay.

| Cytokine | Sensitivity (pg/mL) | Standard Curve Range (pg/mL) |
| --- | --- | --- |
| IL-8 | 1.8 | 8.19~1990.03 |
| VEGF | 2.1 | 13.23~3208.37 |
| IP-10 | 1.18 | 2.59~629.99 |
| PLGF | 3.9 | 11.91~2887.74 |
| IFN-γ | 0.4 | 99.5~24210.49 |
